# Probing nanomechanical, interfacial forces, and surface potential properties of MXene–nanocellulose composites

**DOI:** 10.1039/d5ra07442h

**Published:** 2025-12-01

**Authors:** Jing Li

**Affiliations:** a Department of Chemistry, Stockholm University Stockholm 10691 Sweden jing.li@su.se

## Abstract

The nanomechanical properties, interaction force origins, and surface contact potentials of poly(ionic liquid) (PIL)-modified layered MXenes (Ti_3_C_2_T_*x*_), assembled with holocellulose nanofibers (HCNFs) *via* solution-phase methods, were investigated using PeakForce Quantitative Nanomechanical Mapping (PFQNM) and Kelvin Probe Force Microscopy (KPFM). PFQNM results revealed that incorporation of HCNFs enhanced the structural uniformity and elastic modulus of the composite. The presence of PIL improved the flexibility of the nanomechanical property, likely due to electrostatic dominating interacting forces in colloidal phase. Force spectroscopy using a HCNF-functionalized colloidal probe demonstrated van der Waals attraction between MXene and HCNF at tip compression, and significant energy dissipation at tip retraction, likely aided by hydration forces from hydrogen bond formation. This dissipation was attributed to deformation of chemical groups on HCNFs during compression. KPFM results showed reduced surface contact potentials in PIL-treated MXenes, indicating improved charge transport properties. This effect is attributed to PIL-induced changes in surface dipolarity through electrostatic interactions between the cationic PIL and hydroxyl groups on the MXene. A further reduction in surface potential was observed in MXene–HCNF composites, likely due to residual functional groups on the HCNFs inducing strong van der Waals attraction toward the PIL-modified MXenes, thereby further altering the surface dipolarity. Overall, this study offers new insights into how nanoscale interaction forces govern the mechanical properties and surface charge behavior of MXene–HCNF composites.

## Abstract 2

The nanomechanical properties, interacting forces and electric surface charge properties of PIL-modified MXenes assembled with HCNFs were studied *via* PFQNM, force spectroscopy using the colloidal probe and KPFM.

## Graphical abstract

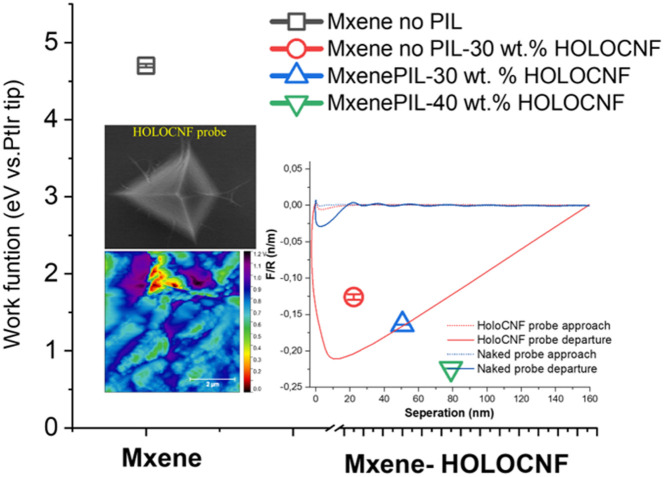

## 1 Introduction

In the realm of functional two-dimensional (2D) materials, MXenes, a family of transition metal carbides, carbonitrides, and nitrides have emerged as next-generation materials for a wide range of applications, exhibiting exceptional properties including high electrical conductivity and mechanical flexibility owing to the tunable surface chemistry of MXenes. To further optimize the surface chemical properties of emerging MXenes, key challenges include developing environmentally friendly synthesis methods, enhancing mechanical strength by controlling the orientation and interlayer spacing of MXene films while maintaining their inherent elasticity, improving oxidation resistance particularly in aqueous environments, and increasing their electrochemical activity.^1^ Future challenges in precise controlling over surface chemistry of MXene include a deeper understanding of surface charge storage and transfer mechanisms in aqueous environments. Surface engineering approaches strongly influence the surface chemistry and their conductivity by altering Fermi level of MXenes. Oxygen (O) terminations consistently increase the work function (*Φ*) relative to the bare surface, hydroxyl (OH) terminations always decrease it, while fluorine (F) terminations can either increase or decrease the work function, depending on the specific MXene material.^2^ However, the poor mechanical flexibility and instability of traditional MXene in air have hindered its development for next-generation electromagnetic interference shielding materials. One strategy to enhance MXene's mechanical properties is to design unique cellular-structured composites by incorporating polymeric fibrils derived from renewable resources, such as cellulose nanofibers (CNFs). The MXene/nanocellulose materials have been used for a wide range of applications in the fields of flexible sensing electromagnetic-interference shielding, pressure sensors as well as wearable health monitoring.^3,4^

As for development of controlled structure of Mxene layers, Poly(ionic liquid) (PIL) has been used as a surface engineering modifier for assemble of MXene laminates *via* electrostatic interactions of PIL modified MXenes with desired properties such as promising catalytic properties and good mechanical properties for high tech electromagnetic devices.^5^ To enhance the mechanical flexibility of MXenes, newly developed cationic PIL-modified layered MXene (Ti_3_C_2_T_*x*_) exhibited excellent colloidal stability during liquid phase synthesis, high flexibility and bending performance for actuator applications. Wide-angle X-ray scattering results revealed that the incorporation of PIL led to varying degrees of intercalation into the MXene nanosheet active layers, resulting in a gradient structure during colloidal-phase preparation. This process enabled the customization of surface properties due to the PIL excellent dehydration performance in acidic conditions, surpassing those of pristine MXenes.^6,7^ Holocellulose nanofibers (HCNFs, denoted as HOLOCNF in the figures) produced from mildly peracetic acid delignified wood fibers have garnered significant research interest for large-scale applications of nanomaterials in the field of 2D materials for developing bio-based eco-friendly composites with excellent mechanical properties and structural flexibility for surface chemical functionalization.^8,9^ Atomic Force Microscopy (AFM) force spectroscopy using nanocellulose-modified colloidal probes have been developed to investigate the adhesion force interactions for hemicelluloses-preserved eco-friendly HCNFs. Interaction force results obtained from force spectroscopy highlighted the significance of the high hemicellulose content of HCNFs, showcasing their high colloidal stability and promising potential for large-scale applications.^8^ A remarkable mechanical strength of 340 MPa was achieved using conventional mechanical testing methods for a blend of aqueous dispersions of MXene (Ti_3_C_2_T_*x*_) and cellulose nanofibrils. This strength was over an order of magnitude higher than the pristine MXene film with 29 MPa. AFM morphological and force spectroscopy results demonstrated that the high strength of the composite was attributed to attractive forces between the two particles in water and further geometric entanglement between them.^10^

To date, however, the mechanisms governing the interactions at small scale between MXene and nanocellulose remain poorly understood. Notably, the degree of dispersion of 2D materials within the CNF matrix is critical for achieving optimal colloidal stability during synthesis.^11,12^ This highlights the need for further investigation into the optimal component ratios by understanding the interaction dynamics between MXene and CNFs in real time experimental conditions. Limited attention has been given to nanomechanical studies using quantitative scanning probe microscopic techniques to assess the mechanical properties of emerging materials. In particular, the localized nanomechanical behavior and nanoscaled interacting forces of CNFs during their interaction with MXenes under liquid-phase, in *operando* conditions remain largely unexplored.

Indeed, Scanning probe microscopy (SPM) techniques, such as Atomic Force Microscopy (AFM), Kelvin Probe Force Microscopy (KPFM), and force spectroscopy have significantly advanced our understanding of the complex relationships among intramolecular forces and their origins explained by Derjaguin–Landau–Verwey–Overbeek (DLVO), nanoscaled mechanical properties, and functional performance in nanomaterials and their composites.^13–18^ Localized nanomechanical information obtained through *in situ* quantitative PFQNM mapping in liquid conditions provided valuable insights into studying the properties of novel 2D and cellulose nanomaterials.^19–21^ Therefore, using SPM enables a deeper understanding of the intermolecular forces and electrical interactions between MXenes and various substrates, which is essential for enhancing the performance of MXene-based devices across a wide range of applications.

Similarly, surface electrical properties such as surface contact potential have rarely been studied using KPFM. Our previous abstract reported that PFQNM measurements on an MXene composite containing 30 wt% HCNFs without PIL treatment showed enhanced nanomechanical properties compared to pristine MXene. The KPFM surface contact potential (or contact potential difference, CPD), which represents a relative value of the surface's work function (*Φ*), was used to visualize the surface charge distribution of the MXene surface.^22^ However, the effect of PILs on the mechanical properties of MXenes, as well as the optimal HCNF ratio for maintaining desirable nanomechanical performance in MXene–nanocellulose composites, requires further investigation.

In this study, PFQNM and amplitude-modulated KPFM were employed to characterize the nanostructure and dispersion of HCNF fibers at two different ratios within both unmodified and PIL surface-modified MXene films, as well as to evaluate the nanomechanical properties of the resulting materials. A HCNF-functionalized colloidal probe was developed to directly measure the adhesive forces between HCNFs and MXene surfaces. The primary objective was to quantify how HCNFs and MXenes influence each other's nanomechanical and intermolecular force behaviors, and to investigate the role of surface contact potential (work function) in these interactions at the nanoscale using KPFM. This study provides new insights into the structure–function relationship of functionalized MXene composites, with a particular focus on localized interactions at the single-fibril and nanoscale levels. A deeper understanding of the nanoscale mechanisms governing both mechanical and surface potential properties offered high-resolution insights and support the development of novel MXene functionalization strategies.

## 2 Experimental

### 2.1. Materials

Experimental details regarding the materials, the synthesis of poly(1-cyanomethyl-3-vinylimidazolium bromide) (PIL cross-linker) modified Ti_3_C_2_T_*x*_ (T_*x*_ represents surface functional terminating groups typically –O, –OH, –F, –Cl^−^) *via* layer delamination method. The bonding between the Mxenes are titanium (Ti), carbon (C), oxygen (O), and fluorine (F) elements, they are uniform distributed. The preparation of MXene-HCNF composites, as well as the characterization of their material properties, can be found in the works cited in the ref. 6, 23, 24 and in SI.

### 2.2. PeakForce quantitative nanomechanical mapping (PFQNM)

Thin films of HCNFs, MXene, and MXene–HCNF composites were mounted onto metallic discs using double-sided adhesive tape. Samples were stored under ambient conditions prior to PFQNM analysis. Measurements were performed using a MultiMode AFM (Bruker, Santa Barbara, USA) equipped with a MultiMode 8-HR controller and an enhanced PeakForce QNM-HR module. Imaging was carried out in PeakForce Tapping for morphology and PeakForce Quantitative Nanomechanical Mapping (PFQNM) modes using ScanAsyst-Air silicon nitride AFM probes (Bruker) with a nominal spring constant of 0.4 N m^−1^ and a tip radius of approximately 2.0 nm. Before use, probes were treated with a UV ozone cleaner (ProCleaner Plus) for ∼20 minutes. Prior to each PFQNM measurement, calibration of the optical lever sensitivity was conducted by indenting the cantilever against a hard sapphire surface (SAPPHIRE-12 M in Bruker PFQNM calibration kit), using the quasi-static force method. The spring constant of each cantilever was independently calibrated using the thermal tune method, consistently yielding values around 0.3 N m^−1^.^25,26^ To ensure consistent and reliable force measurements, the indentation depth was carefully controlled throughout each scan. A PeakForce setpoint of 300 piconewtons (pN), a scan rate of 0.8 Hz, and a maximum image resolution of 512 × 512 pixels were used for all PFQNM acquisitions.

### 2.3. Kelvin probe force microscopy measurements and probe calibration

Surface contact potential mapping was carried out using amplitude-modulated Kelvin Probe Force Microscopy (KPFM) on a MultiMode AFM (Bruker, USA) equipped with a MAC III controller, enabling multi-frequency measurements. Both surface topography and contact potential were simultaneously recorded in single-pass mode to ensure high lateral resolution. Topographical data were acquired at the cantilever's resonance frequency (∼80 kHz), while Volta potential signals were collected at a much lower frequency (∼2 kHz) to minimize signal interference and cross-talk between the two channels. For KPFM measurements, PPP-EFM probes with a PtIr5 conductive coating (NanoAndMore USA) were used. These probes feature a resonance frequency of approximately 75 kHz and a spring constant of 2.8 N m^−1^, making them suitable for high-resolution electrical characterization. KPFM imaging experiments were conducted on specimens mounted on conductive stainless-steel discs in ambient air under controlled humidity, in order to minimize temporal drifts in surface contact potential (CPD). Temporal variations in CPD were continuously monitored across the same scanning area.

Special attention was given to interfacial regions between MXene and HCNF, where selected areas were mapped to explore surface potential distribution and heterogeneity at the nanoscale. The absolute work function value can be obtained if the work function of the probe is known, which requires calibration using a reference sample with known work function values.^27^ In this study, a two-component Au–Al reference sample (Bruker calibration kit) was used to calibrate the work function (*Φ*) of the probe. The known work functions of the reference metals are 4.82 eV for Au and 4.06 eV for aluminum, respectively. The measurements were conducted using a PPP-EFM probe (NanoAndMore), coated with 25 nm thick PtIr5 and has a resonance frequency of 75 kHz. In the KPFM experimental setup, the compensation bias is applied to the PrIr coated probe, and consequently, the probe's *Φ* can be calculated as KPFM measured a CPD that can be expressed as: |*e*|*V*_CPD_ = *Φ*_probe_ – *Φ*_sample_. Where, the *V*_Probe_ (CPD) is the compensation bias applied on probe, *Φ*_Probe_ is the PtIr probe work function, *Φ*_Sample_ represents the reference sample's theoretical *Φ*, *e* is the elementary charge (1.602 × 10^−19^ C). The calibrated work function (*Φ*) of the Pt/Ir probe was calculated using the measured contact potential difference values for each component the Au and Al. The resulting *Φ*_probe_ (_PtIr_) *vs.* Au. is 5.49 ± 0.0015 eV, *Φ*_probe_ (_PtIr_) *vs.* Al is 4.77 ± 0.0092 eV, this is agreed with previous report.^27^ The KPFM images in SI-Fig. 4 showed typical maps of *Z*-height and CPD obtained for the Au–Al reference sample using the PtIr probe.

### 2.4. Scanning electron microscopy

Scanning electron microscopy (SEM) analysis was carried out using a JEOL JSM-7000F (Japan) to examine both unmodified ScanAsyst-air AFM probes and those functionalized with HCNF, before and after PFQNM measurements. To maintain the structural integrity of the nanocellulose layer, the probes were mounted to conductive carbon tape without any additional coatings. Imaging was performed at an accelerating voltage of 5 kV and a working distance of 19 mm. After PFQNM testing, SEM imaging was repeated to assess the probes for tip damage or potential surface contamination.

### 2.5. Colloidal probe functionalization by HCNFs and force spectroscopy

**Fig. 1:**
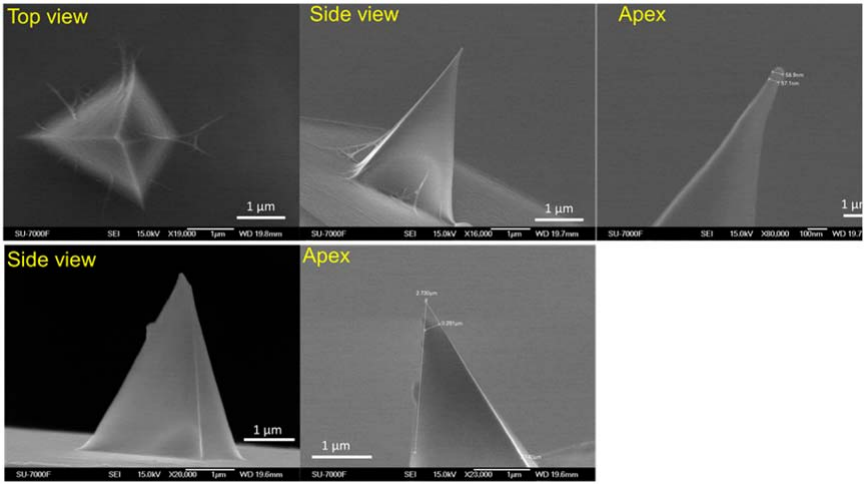
Representative SEM images of the colloidal probes: (upper panel) top, side, and apex views of the HCNF-modified colloidal probe; (bottom panel) side and apex views of a used unmodified (naked) probe.

HCNF fiber modified colloidal probe was made using the sharp silicon nitride probes (Bruker ScanAsyst-air), and high molecular weight of polyethylenimine (PEI) (Sigma) was used as a pre-coat. Attaching of HCNFs (suspension concentration was 0.12 wt%) was carried out using dip-coat layer-by-layer (LBL) method in a highly controlled manner. In short, HCNFs were coated on the pr-coated tip surface using LBL dipping assembly of PEI (2.5 g L^−1^) for 10 min and then HCNF (0.12 g L ^−1^) for 15 min, with a washing step with Milli-Q water in between (3 minutes contact in each) to form one bi-layer.^28,29^Fig. 1 below shows SEM images of (a) the top, side, and apex views of a HCNF-coated AFM probe, and (b) the side and apex views of a used uncoated (naked) probe. Clearly, HCNF fibers are uniformly attached to the apex region of the coated tip. The HCNFs are also evenly distributed across the entire AFM tip, forming a monolayer. The thickness of the HCNF monolayer is approximately 20 nm.

Representative SEM images of the used nanofiber probe (SI-Fig. S1) show that the nanocellulose coating remained firmly attached even after multiple PFQNM measurements, demonstrating both the high stability and rigidity of the coating at the AFM tip, as well as the reliability of the layer-by-layer (LBL) method developed in this work. All force–distance (FD) measurements were conducted in triplicate, using non-modified probes as controls and nanocellulose-modified ScanAsyst-fluid probes. The probe approach speed toward the film surfaces was set to 0.5 Hz in all cases. Each force map recorded a maximum resolution of 512 × 512 pixels, yielding over 60 000 force–distance curves per complete mapping session. Notably, the force curves obtained under a given set of experimental conditions were highly reproducible across repeated compression cycles, with all curves following consistent trends. SI-Fig. S2 presents over 500 force–distance curves measured from two different areas of the same MXene film, using both unmodified and modified probes.

### 2.6. Data analysis

Analysis for maps of PFQNM and KPFM images included the top surfaces of MXene films, the tops of nanocellulose fibers, and the edge areas where MXene is embedded within the HCNF fiber matrix. Measurements were conducted in duplicate for each component. Initial image processing was performed using NanoScope analysis software. To correct for height inconsistencies and surface curvature, small-scale height images were flattened using a second-order polynomial fit. This flattening process removed *Z*-offsets between scan lines and corrected for tilt and bow across the image. A least-squares fit was applied to the unmasked portions of each scan line and subtracted, effectively eliminating large-scale curvature and enhancing the visibility of fine surface features. Quantitative PFQNM maps were analysized from selected regions of interest. Data analysis for the maps of *Z*-height, elastic modulus using Derjaguin–Muller–Toporov (DMT) mechanical contact model, adhesion force, and surface contact potential was done using at least five images per specimen type, with scanning areas ranging from 1 µm × 1 µm to 5 µm × 5 µm. All measurements were performed in duplicate to ensure reproducibility.

For nanomechanical data interpretation, the force acting on the cantilever relative to the adhesion force during PFQNM measurements is described by eqn (1):1
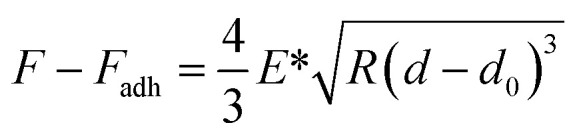
2
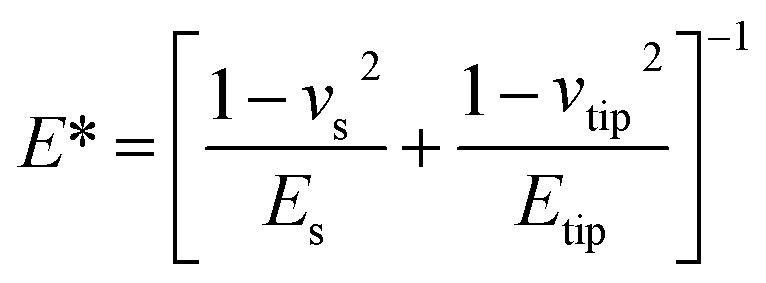
where, *F – F*_adh_ is the applied force minus the adhesion force, *R* is the tip radius, and *d – d*_0_ represents the sample deformation. The reduced modulus (E*) obtained from fitting can be used to calculate the sample's Young's modulus (*E*_s_) when the Poisson's ratio is known, using eqn (2): where *ν*_s_ and *E*_s_ are the Poisson's ratio and Young's modulus of the sample, and *ν*_tip_ and *E*_tip_ are those of the AFM tip.3
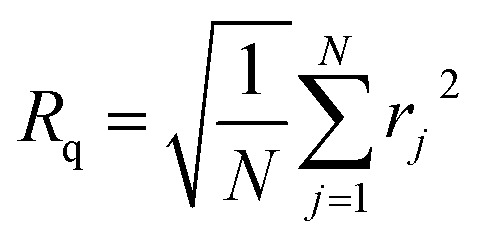


Surface roughness was evaluated through statistical analysis by selecting regions of 500 nm × 500 nm from each AFM image. The root mean square (RMS) roughness, denoted as *R*q, represents the average of the squared deviations from the mean height within the selected area. It was calculated using eqn (3), as described in our previous studies.

Fig. S3 below showcased representative fitting process for obtaining height distribution and modulus distribution of the PFQNM maps of films.

For each image of PFQNM and KPFM, multiple regions within individual channels were selected for analysis. To perform a detailed statistical evaluation of modulus, adhesion force, and surface contact potential distributions, we utilized the height distribution functions available in the Gwyddion freeware. Gaussian fitting was applied to achieve the best statistical representation of the SPM data. During the fitting process, normalization of the density functions *ρ*(*p*)—where *p* represents the measured parameter (*e.g.*, modulus, force, or potential)—was carried out using the function: *f*(*x*) = *y*_0_ + *a*·exp[–(*x* – *x*_0_)^2^/*b*^2^], as implemented in the software. These statistical functions are generated as normalized histograms of quantities such as height, force, or modulus, which are calculated as directional derivatives (either horizontal or vertical) within the selected image area. The corresponding cumulative distributions are integrals of these densities and range from 0 to 1, ensuring that the results are independent of both the number of measured data points and the number of histogram bins.^14,30^

**Fig. 2:**
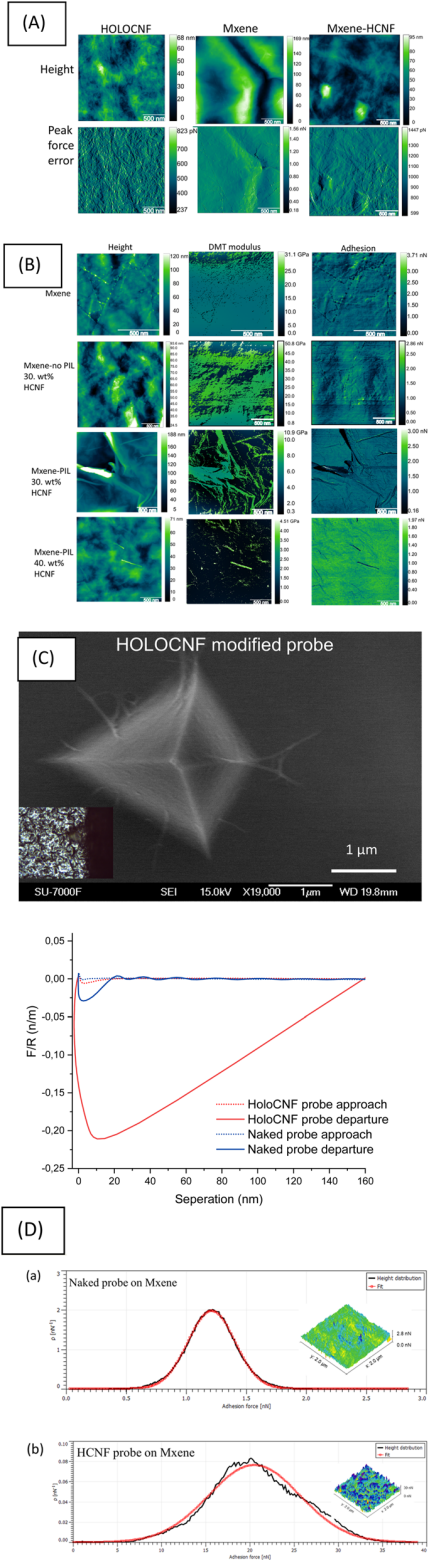
Representative results showing: (A) Peakforce tapping AFM *Z*-height image and PeakForce error; (B) PFQNM maps of *Z*-height, DMT modulus, and adhesion force; (C) SEM image of the HCNF-modified probe; representative normalized force–distance curves for Mxene (red curves were measured using the HCNF-modified probe); (D) Gaussian-fitted adhesion force distributions for HCNF–Mxene and pristine Mxene surfaces. (Note: results of PFQNM for MXene and MXene no PIL-30 wt% HCNF are reused from our abstract.^22^

The errors for the statistical values reported for each calculated quantity (*i.e.*, *Z*-height, adhesion, modulus, surface contact potential) are determined as follows according to method used our previous work.^15^ Step (1) for one area of a single image, the error is calculated based on the peak values from the normalized density functions *ρ*(*p*), where *p* is the corresponding quantity (*i.e. Z*-height, adhesion, modulus, or surface contact potential). These peak values are derived using the one-dimensional autocorrelation function, which is commonly assumed to follow a Gaussian form. In SPM measurements, the Gaussian autocorrelation function generally provides a good approximation of the surface properties. Step (2) the overall average error was calculated from five error values. The five error values were obtained from five different areas on one SPM image, each error was calculated according to the method as described in Step (1). Step (3) at least two specimens of each sample type were used to acquire SPM images. Therefore, the final reported error for the peak value of a given quantity's density was calculated as the average of the peak density values obtained from measurements on the two specimens. The DMT model accounting for adhesion force outside the tip indented area, is well-suited for materials with high stiffness, low adhesion, and small indentation, making it appropriate model for nanocellulose-2D composite materials. It is important to note that the absolute modulus values are significantly influenced by the chosen contact mechanics model, tip geometry, and other imaging parameters.^19,26^ Therefore, it is important to note that the modulus values derived from these measurements are intended for comparative purposes only. They should be interpreted in terms of relative variations, which allows for meaningful discussion of the stiffening or softening effects resulting from HCNF incorporation into MXene matrices, and *vice versa*. The average tip radii, measured by multiple times of SEM experiments, were 51.1 nm, 118.0 nm, and 121.0 nm, and were used to fit the PFQNM elastic modulus data for the pristine MXene film and the three types of MXene–HCNF composite films (with and without PIL treatment), respectively. Based on SEM analysis, the averaged tip radii of the used uncoated and coated probes were approximately 51.1 nm and 121 nm, respectively. These values were used to normalize the force (F/R) for the force–distance curves shown in Fig. 2C. The low PeakForce setpoint of 300 pN was used during measurements was to minimize potential tip wear, particularly given the non-uniform and blended nature of the MXene–HCNF composite surfaces. Uniform imaging parameters were consistentlyapplied across all PFQNM measurements to ensure the reliability and comparability of the data.^21,31^

## 3 Results and discussion

The Peakforce tapping AFM morphology results (Fig. 2A) revealed a uniform distribution of the HCNF nanostructure, with the nanofibers tightly embedded within the thin MXene sheets. The height distribution indicated that the incorporation of HCNFs reduced the surface roughness of the MXene, thereby enhancing the overall uniformity of the composite. Specifically, the surface roughness of the pristine MXene film (1 × 1 µm) was measured at 56.8 nm ± 71 pm, while that of the MXene–HCNF composite was 28.4 nm ± 69 pm. However, the average roughness values from PFQNM obtained from multiple regions of the sample, as shown in Table 1, did not show significant differences. This suggests that the incorporation of HCNFs did not markedly alter the overall surface roughness of MXene, but rather contributed to the formation of a uniform networked composite structure. The elastic modulus of PFQNM determined using the DMT model, reached a maximum of approximately 62.3 GPa ± 40.4 Mpa for the composite containing 30 wt% HCNF without treatment by PILs, compared to approximately 26.7 GPa ± 13 MPa for pristine MXene (Fig. 2B). The Young’s modulus of the composite was therefore approximately two times higher than that of the pure MXene film. As expected, the elastic modulus measured for pure HCNFs was low, approximately 0.2 GPa ± 30 MPa (see SI-Fig. S5).

**Table 1:** Statistical analysis of the DMT modulus, adhesion force, and height distribution obtained from PFQNM measurements

Materials	DMT modulus (Gpa)	Adhesion force (nN)	Height distribution (nm)
MXene	26.7 ± 7.4^a^	1.6 ± 0.3	99.1 ± 51.4
HCNF	0.2 ± 0.03 c	0.7 ± 0.2	17.2 ± 2.7
Mxene noPIL 30wt% HCNF	62.3 ± 40.4^b^	2.3 ± 0.7	89.2 ± 46.4
MxenePIL 30wt% HCNF	17.7 ± 7.9^c^	1.3 ± 0.1	92.8 ± 31.3
MxenePIL 40wt% HCNF	4.3 ± 0.4^c^	1.3 ± 0.0	86.3 ± 36.8

a tip radii 51.1 nm was used for DMT modulus calculation.

b tip radii 118.0 nm was used for DMT modulus calculation.

c tip radii 121.0 nm was used for DMT modulus calculation.

As shown in Table 1, the statistic of DMT elastic modulus of the MXene–HCNF composite containing 30 wt% HCNF decreased after MXene modification with PIL, indicating that PIL likely enhanced the bending flexibility of pristine MXene. However, PIL also appeared to soften the composite overall.

Force–distance (FD) curves measured by PFQNM force spectroscopy in air was employed to quantify the force between HCNF and the MXene surface using a HCNF-modified AFM probe (Fig. 2C and D). The FD curve during tip compression (the dotted red line) showed a DLVO force-liked attraction at a separation distance of approximately 10 nm. The shallow attraction might be suggested that the HCNF probe could have operated in the van der Waals attractive region if in liquid conditons, and cannot overcome the energy barrier to reach the primary minimum at shorter separation distances (<3 nm). This behavior is maybe related to the strong surface charge density on MXenes.^18,32^ The FD curve (the solid red line) measured during tip's retraction (*i.e.* tip departure) (AFM scanner retraction) from the MXene surface revealed a nominalized adhesion force that was 4 times stronger (17.2 nN ± 77 pN) than the adhesion force measured for the same MXene film when a naked probe was used (1.5 nN ± 1.2 pN). These results clearly indicate that the force interaction between nanocelluloses and MXene originated from van der Waals attraction, with adhesion being a long-range force at a separation distance of 150 nm. Energy dissipation (*W*) is given by the force times the velocity integrated over one period of the vibration as described in eqn (4). The dissipation is therefore the hysteresis between the approaching and retraction force curves.^19^4
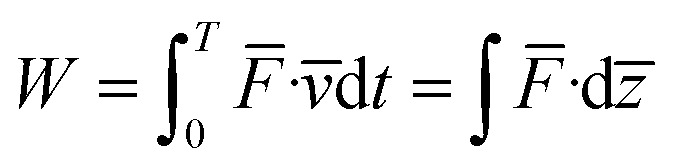


Remarkably, as shown in the representative FD retraction curves and the additional FD curves provided in the SI, the adhesion regions (area below zero force of red solid curve) during tip retraction exhibited significantly greater energy dissipation (*i.e.*, energy loss) compared to that observed with the naked probe (area below zero force solid black curve). The difference in energy dissipation can be attributed to the deformation of the well-preserved cellulose and hemicellulose components without lignins on HCNF monolayer attached to the probe, whereas no such layer was present when the MXene surface was compressed against the bare Si_3_N_4_ surface of the unmodified probe. This observation aligns with previous results.^15,20^ The FD curves unveiled a mechanism of force interaction between the surface of MXene and the surface of HCNF attached to the AFM tip.

Understanding interaction forces between colloidal particles is essential for control of materials surface chemical interactions. DLVO theory describes the surface forces between colloidal particles in liquid as the sum of the van der Waals force and the electric double-layer force. For two identical colloidal particles, the van der Waals force is attractive and the electric double-layer force is repulsive. There is hence a competition between van der Waals attraction and electrostatic repulsion arising from the electrical double layer of counterions. This interplay influences the DLVO interaction energy between charged surfaces of the cationic PIL, Mxenes and nanocelluloses in electrolyte solutions.^7,15,18,33^ During the colloidal phase of MXene interaction with HCNFs, it is possible that the DLVO interaction energy at the Water–HCNF–MXene interface was altered by PIL during the interaction, leading to irreversible attachment at the secondary minimum of the DLVO potential, with the major axis of Ti_3_C_2_T_*x*_ aligned parallel to the HCNF surface. It was likely not possible to overcome the repulsive energy barrier at the primary minimum of the DLVO potential.^32^ As a result, although our FD measurements were conducted under ambient conditions, it can be presumed that the interactions between MXene and HCNF in the presence of PIL involved only repulsive electrostatic forces at the air–HCNF–MXene interface. In contrast, in the absence of PIL, MXene–HCNF interactions likely included van der Waals attractions and possibly hydrodynamic forces. This is further supported by the observation that the average adhesion force for the MXene–HCNF composite without PIL treatment was higher (2.3 ± 0.7 nN) compared to the composite with PIL-treated MXene (1.3 ± 0.1 nN). The changes in adhesion force measured for composites with higher HCNF (40 wt%) content, synthesized using the same amount of PIL, were negligible, indicating that the weight percent of nanocellulose had no significant influence on the adhesion force.

Effects of functional groups and surface charges present in the MXenes have been explained as follows. Because the as synthesized HCNFs are negative charged (charge density 200 microequivalents per gram by conductometric titration).^8^ The pristine Ti_3_C_2_T_*X*_ nanosheets are negatively charged. PIL-Mxene is positively charged.^6^ There is hence strong adhesion between oppositely charged PIL-Mxene (Ti_3_C_2_T_*X*_) and HCNFs during the synthesis in colloidal phases. The nonbonding interactions between different atoms in the system include both electrostatic and van der Waals forces. The formation of the HCNF-intercalated PIL–MXene microstructure can be attributed to two main factors related to surface charge and surface chemical groups: (1) the heterogeneous distribution of glucomannan and xylan the repeating units of the glucomannan on the HCNF-coated nanosheet precursors, which leads to variations in the surface charge distribution of the PIL–MAX flakes; and (2) the positively charged PIL–MXene flakes preferentially stacking on top of the negatively charged HCNFs during the vacuum-assisted filtration step, driven by electrostatic attraction. This interpretation is supported by the zeta potential data provided in SI. As a result, a gradient PIL-intercalated MXene microstructure is formed, with the amount of PIL decreasing from the interface between the two layers toward the outer surface of air/PIL–MXene. Upon interaction with HCNFs, the interlayer expansion at the interface between the HCNF and PIL– MXene layers is greater than in regions further from the interface. This expansion induces rapid bending together with the swelling of PIL- MXene layer during inner bending, ultimately reducing the mechanical strength of the PIL–MXene–HCNF composite compared to the MXene–HCNF composite films.

The revealed origin of the measured interaction forces in air suggests an underlying mechanism for the notable colloidal stability of the HCNF–MXene suspension during synthesis in an aqueous environment. It also provides quantitative insights into the strength of the attractive forces involved when MXene interacts with nanocellulose under colloidal-phase experimental conditions. The strong adhesion between HCNFs and MXene is attributed to the various chemical groups uniformly distributed across the nanocellulose surface, which were preserved during the mild HCNF synthesis. This results in strong adhesion during tip compression, and high energy dissipation (damping) as the result of inelastic CNF deformation during tip retraction (*i.e.* CNF probe lift off from Mxene surfaces). However, additional investigations using the force spectroscopy under liquid conditions are necessary to validate these proposed explanations.

**Fig. 3:**
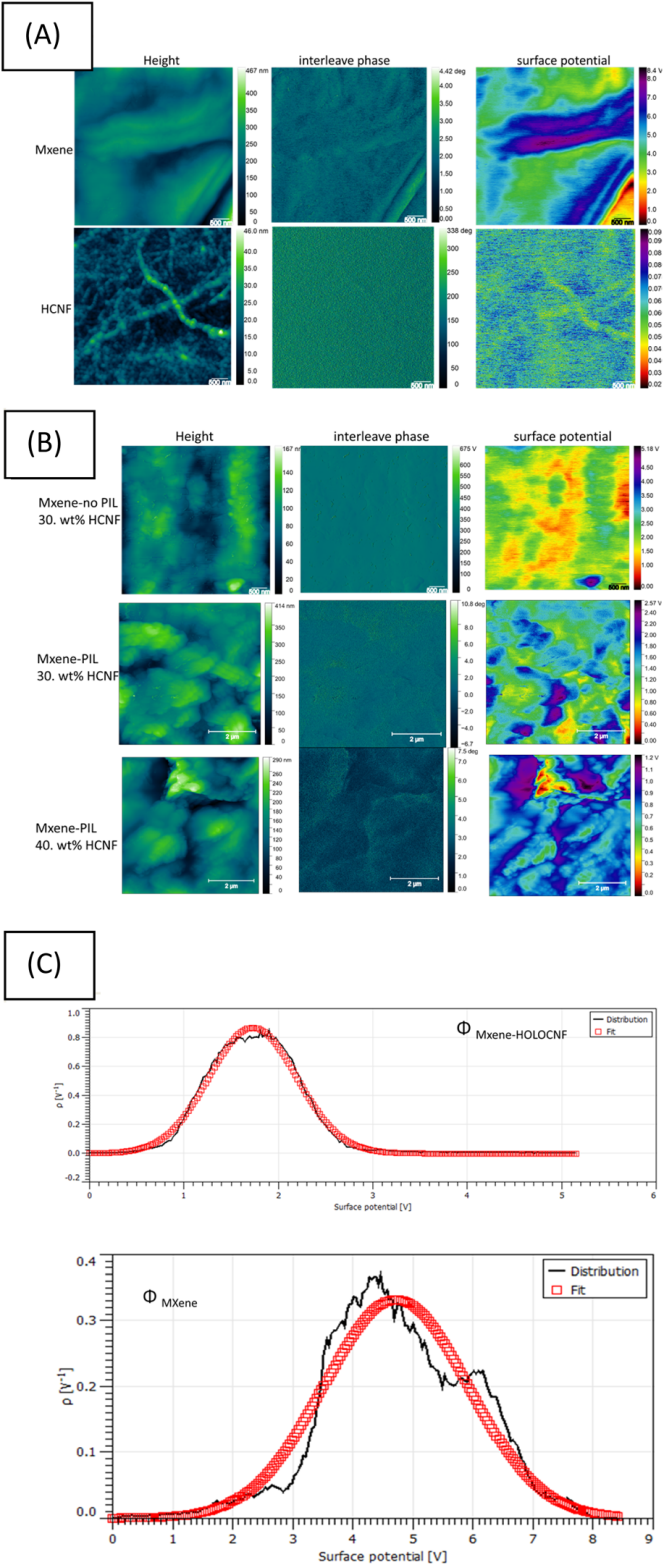
(A) Representative amplitude-modulated KPFM images showing *Z*-height, interleave phase, and surface potential for the three materials: HCNF, pristine MXene. (B) Surface potential maps measured for three composites: the HCNF–MXene composite without PIL treatment, the PIL treated MXenes containing 30 wt% and 40 wt% HCNF, (C) Gaussian-fitted results of the work function for MXene and the HCNF–MXene composite (MXenes without PIL treatment).

The calibrated PtIr KPFM probe was used to map the local variation of the absolute CPD over the Mxene and its composites. As shown in Fig. 3 below, the localized heterogeneous electrical reaction measured by a Pt\Ir probe occurring at the active sites of single-layer MXene and the residual chemical groups on HCNF were *in situ* quantified and visualized by KPFM. The surface contact potential (*Φ*) of MXene decreased from 4.7 eV to 1.7 eV after interaction with PIL cross linkers and HCNFs in colloidal chemical phase.

**Fig. 4:**
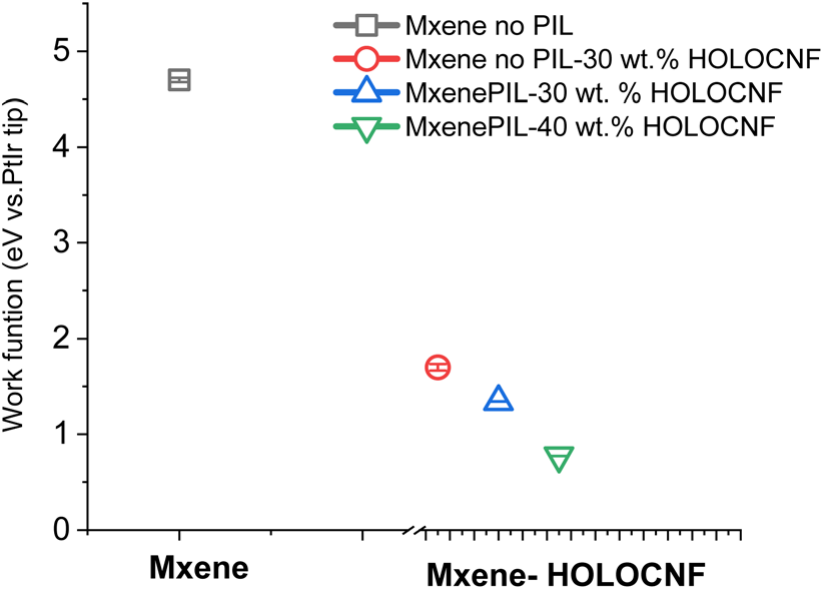
Statistical analysis of the surface potentials for Mxene and the three types of MXene–HCNF composites, (note: KPFM results are reused from our abstract).^22^

The absolute *Φ* values were reported in Fig. 4 and the Table in SI-S4. Maps of the work function (middle panel in Fig. 3) and the corresponding statistical analysis (bottom panel in Fig. 4) revealed that the work function of pristine MXene (Ti_3_C_2_T_*x*_), denoted as *Φ* MXene was measured to be 4.7 eV (±22 meV). This value is attributed to the presence of – O, or –OH surface termination groups. The results are consistent with previously reported values for types of Ti_3_C_2_T_*x*_ with –OH, Cl^−^ terminations.^34^ Upon incorporation of 30 wt% HCNF, the work function of the resulting MXene–HCNF composite (*Φ*_MXene-HCNF_) decreased to 1.7 eV (±34 m eV), even in the absence of PIL cross-linkers during synthesis. When PILs were included during synthesis, the surface potential of the same composite was further reduced to 1.3 eV. The reduced work function of MXenes upon interaction with PIL can be attributed to the gradient structure introduced by PIL intercalation into the MXene active layers, which modulates surface charge distribution.^6^ This modification of charge distribution results in lowered Schottky barrier heights, potentially minimizing Fermi level pinning at conductive interfaces of Mxene layers. This phenomenon arises from the formation of surface dipoles induced by PIL cationic molecules. Combined with weak Fermi level pinning, these dipoles facilitate Schottky-barrier-free hole or electron injection into the PIL modified MXenes *via* van der Waals junctions, particularly on O-terminated or OH-terminated MXene surfaces.^35^ Because charge transport in Ti_3_C_2_T_*x*_ layers is band-like, the intralayer transport is enhanced by the PIL-induced alteration of the out-of-plane orientation of MXene multilayers.^36^ As a result, the electron transfer rate across the MXene surface may increase due to the modified charge transport behavior.^2,37^ The PIL-modified MXenes reported here offer tunable surface polarity and enhanced conductivity, making them strong candidates for applications in green energy storage.

Additionally, increasing the concentration of HCNF led to a further decrease in the surface potential of MXene, reaching as low as 0.8 eV. This significant reduction of surface contact potential suggests a doping mechanism involving the formation of Ti–O⋯H– hydrogen bonds between MXene and the chemical groups on HCNFs. This interaction maybe modulated the dipolarity of the Mxene surface, thereby lowering its work function. These findings are consistent with previous reports.^38^

## 4 Conclusions

In this work, a combination of PeakForce quantitative nanomechanical (QNM) AFM mapping and force spectroscopy using a newly developed nanocellulose colloidal probe and the KPFM was developed to study nanoscale mechanical properties, the origins of interaction forces and surface charge distribution between HCNFs, MXene, and PIL-modified MXene. Our results revealed a clear relationship between the structure, nanomechanical behavior, and surface chemical and electrochemical properties of MXene–HCNF composites at the nanoscale. The key findings are:

• For the first time, PFQNM results revealed that incorporating HCNFs significantly enhanced the nanomechanical properties of MXenes compared to pristine MXene or HCNF alone. The increased elastic modulus of the composite highlights the potential of using eco-friendly HCNFs as a reinforcing matrix to improve the flexible mechanical performance of MXene-based 2D materials, offering a sustainable alternative to fossil-derived nanomaterials. The presence of PIL improved the flexibility of the mechanical properties of MXene–HCNF composites, maybe attributable to interacting repulsion force during reactions in the liquid colloidal phase.

• For the first time, KPFM imaging visualized the surface contact potential distributions at the heterointerfaces of the composites. The work function (*Φ vs.* Pt/Ir probe) of pristine MXene (∼4.7 eV) was significantly reduced after interaction with HCNFs and PILs, indicating that these interactions modulate the surface dipolarity due to an altered surface charge distribution induced by PIL cations. This reduction in work function suggests improved electrical charge transport properties of MXene following PIL treatment. The enhancement is attributed to electrostatic complexation by the PILs and intermolecular interactions between the PIL and residual chemical groups on the HCNFs. KPFM results suggested that the incorporation of PIL into the MXene colloids lowered the energy barrier of electron transport between surface groups (*i.e.* —Ti, –C, –O, –F) of the MXene layers, thereby enhancing charge-transfer efficiency.

• Force spectroscopy results using a HCNF-functionalized colloidal probe demonstrated strong van der Waals attractions between HCNFs and MXenes at tip compression. Hydration forces may also contribute, likely due to the formation of Ti–O⋯H–N hydrogen bonds. Significantly higher energy dissipation was observed during tip retraction at HCNF–MXene interfaces compared to pull-off force with a bare AFM probe. This dissipation is attributed to the deformation of chemical groups on HCNFs during tip compression. The force spectroscopy data further demonstrated that competition between electrostatic and van der Waals (vdW) forces, arising from shifts in the relative energy balance of these two interactions affected on the nanomechanical properties of the Mxene-HCNF composites.

• The presumed mechanism of the interaction forces, based on DLVO theory, is that the incorporation of PIL modifies the electrostatic forces occurring at the MXene interfaces. The interactions between HCNFs and PIL-modified MXenes significantly alter the dipolar characteristics and surface charge distribution of the MXene surface. These findings also explain the excellent colloidal stability of HCNF–MXene suspensions and indicate that the strong adhesive forces originate from the diverse functional groups retained on HCNFs after mild processing.

Overall, this study provides a comprehensive understanding of the structure–function relationship in PIL–MXene–HCNF composites, illustrating how nanomechanical, surface chemical, and surface charge properties emerge from nanoscale intermolecular force interactions. The findings offer new insights into the intermolecular interaction mechanisms between 2D materials and nanocellulose, contributing to the development of next-generation functional materials.

## Author contributions

Jing Li: conceptualization, methodology, writing – original draft preparation. Writing – reviewing and editing, data curation, software, validation, visualization, investigation. Supervision.

## Conflicts of interest

The author declares that they have no known competing financial interests or personal relationships that could have appeared to influence the work reported in this paper.

## Supplementary Material

RA-015-D5RA07442H-s001

## Data Availability

All relevant data supporting the findings of this work are available within the article and the supplementary information (SI). Supplementary information is available. See DOI: https://doi.org/10.1039/d5ra07442h.
