# Supplementary material for: Probing nanomechanical, interfacial forces, and surface potential properties of MXene–nanocellulose composites
Source: RSC Adv. 2025 Dec 1;15(55):47291–9. doi: 10.1039/d5ra07442h (PMC12666778; doi:10.1039/d5ra07442h)
Supplement: RA-015-D5RA07442H-s001 [file RA-015-D5RA07442H-s001.pdf]

Supplementary Information (SI) for RSC Advances.
This journal is © The Royal Society of Chemistry 2025

Supporting information (SI)

Probing nanomechanical, interfacial forces, and surface potential properties of MXene–Nanocellulose composites

Jing Li*[a]

Department of Chemistry, Stockholm University, Stockholm, 10691, Sweden

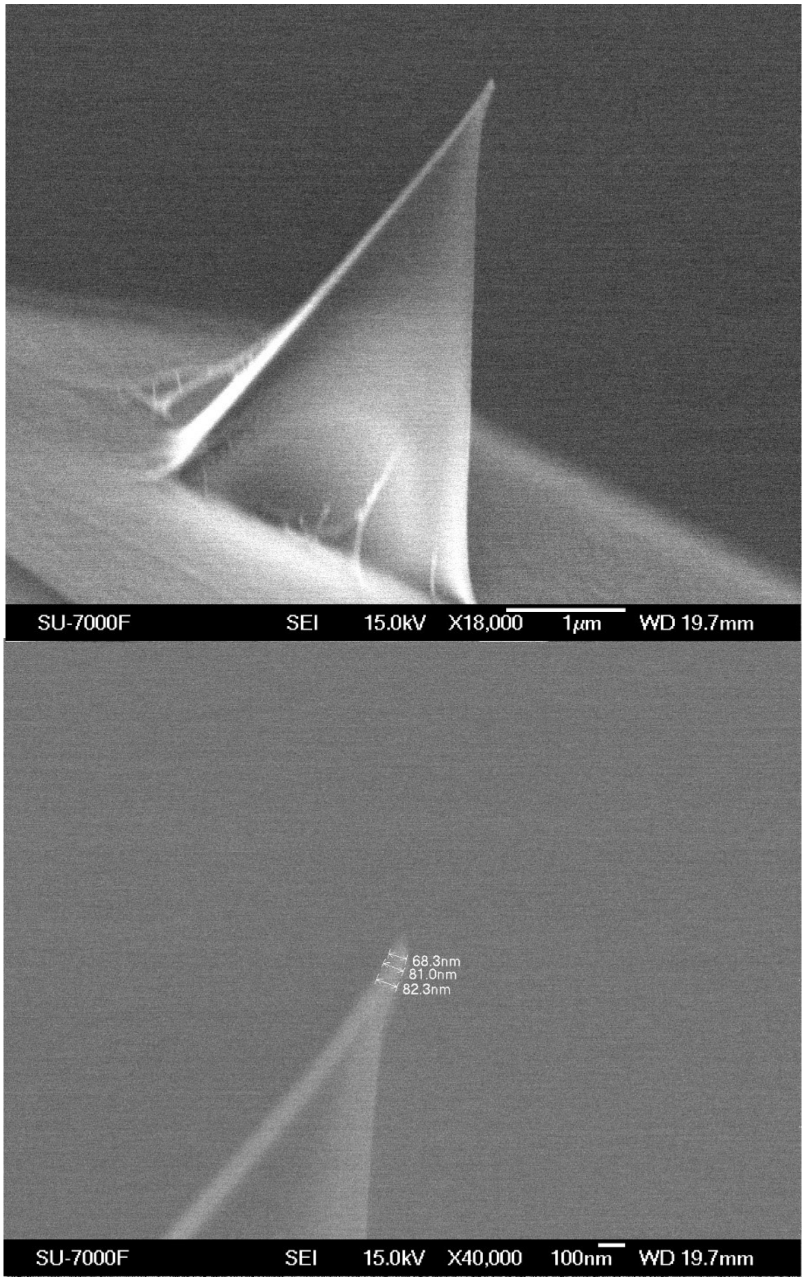

Figure S1. Representative SEM images of the used HCNF colloidal probe showed that the nanocellulose coating remained firmly attached even after multiple PFQNM measurements.

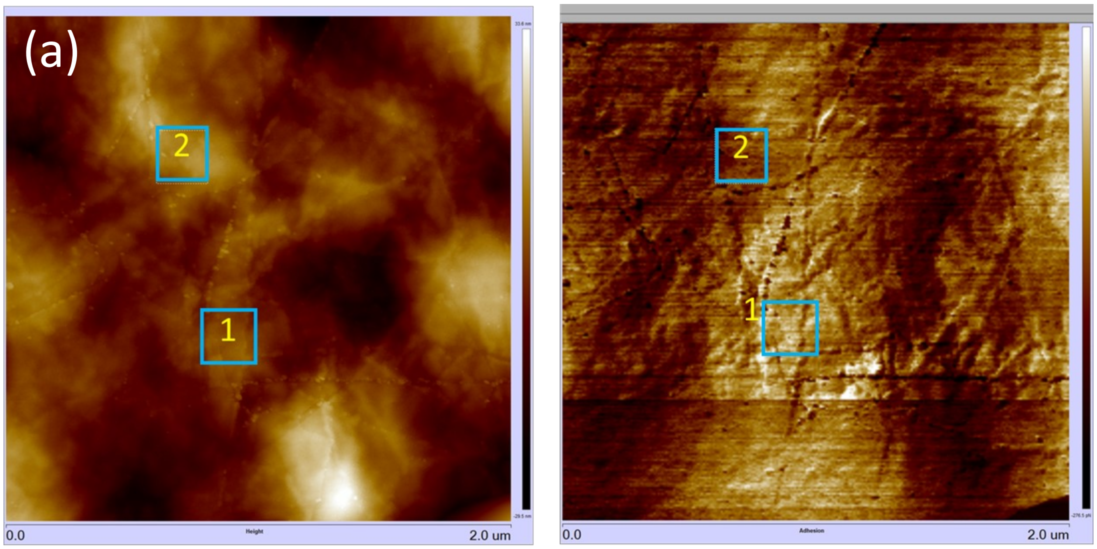

Height Adhesion

FD curves measured for area 1

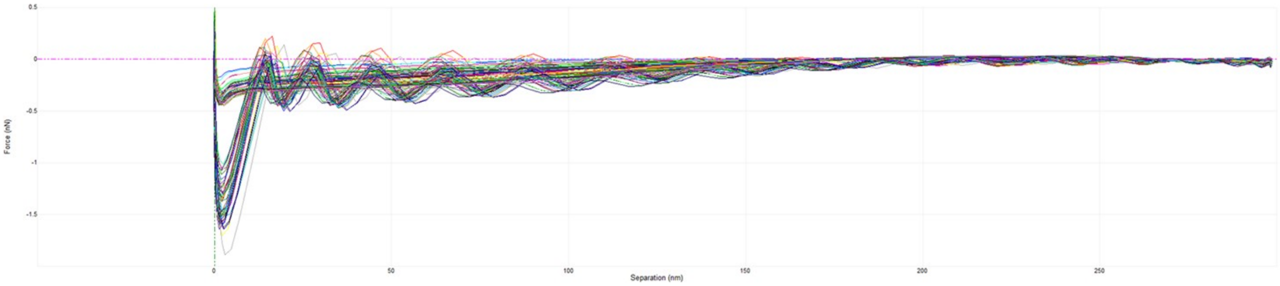

FD curves measured for area 2

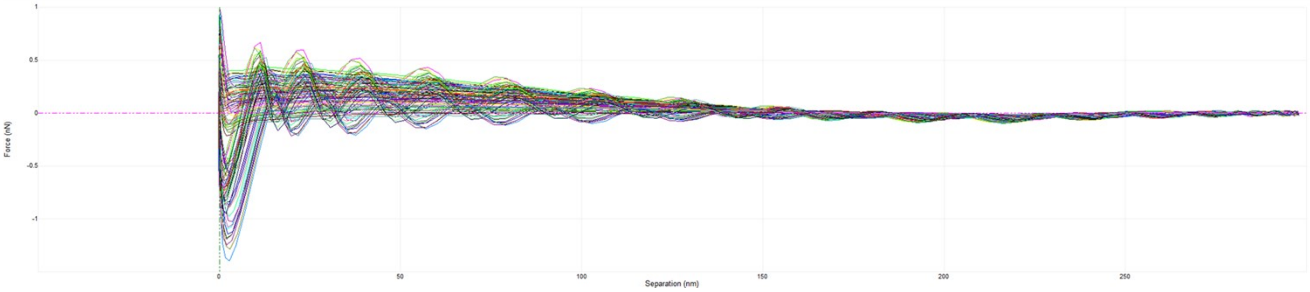

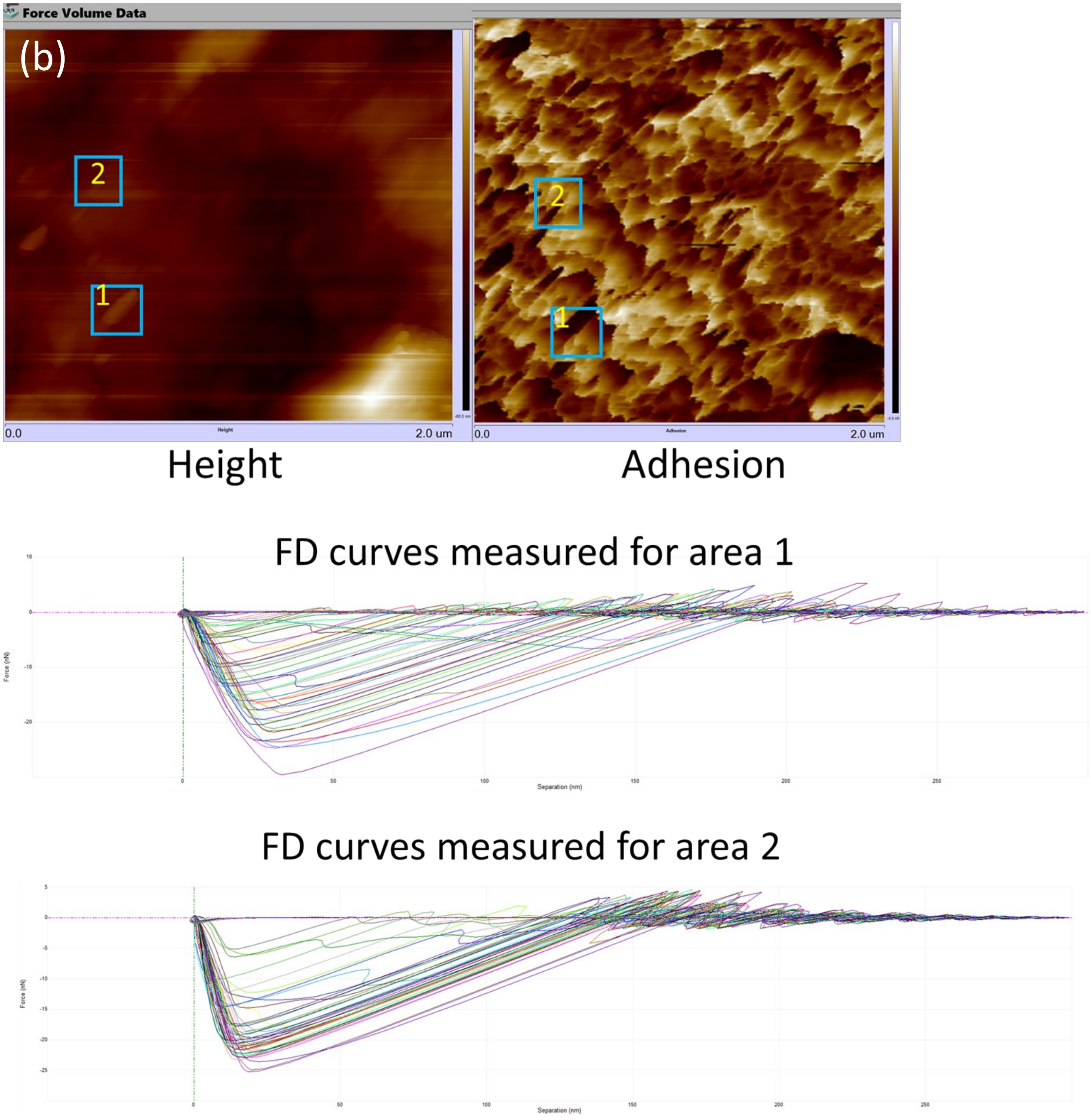

Figure S2. Representative force distance curves (> 500) measured for two areas of (a) the Mxene film using naked probe, (b) the Mxene film using HCNF modified probe.

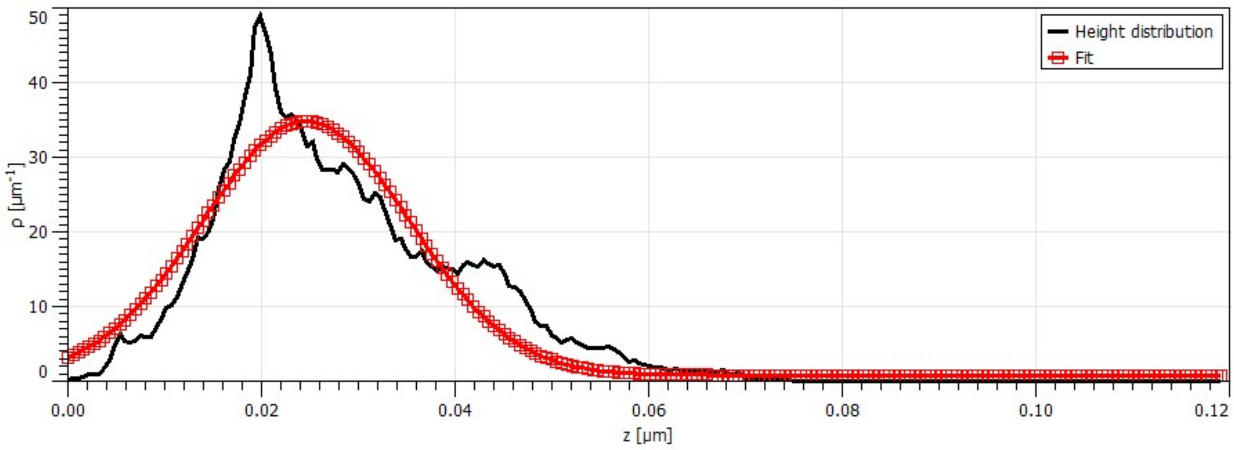

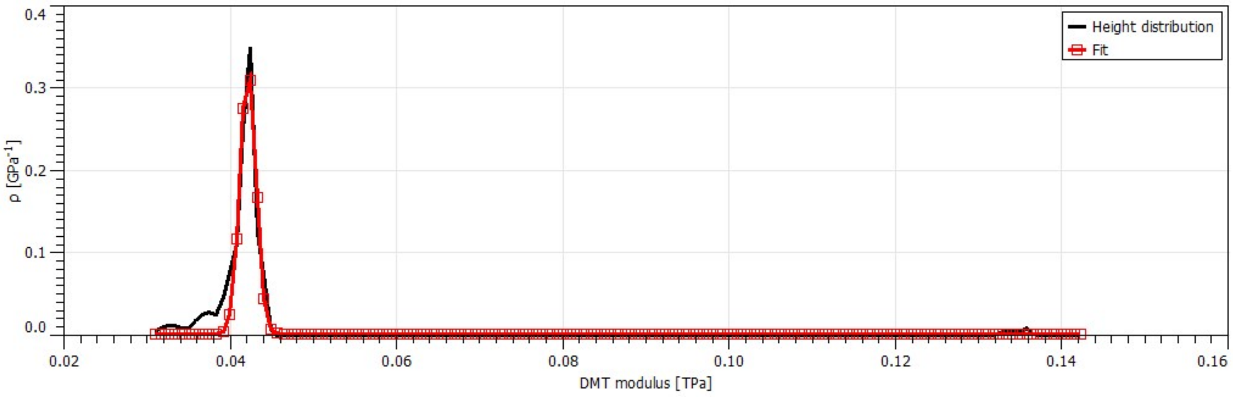

===== Fit Results =====
Data: Height distribution
Number of points: 137 of 137
X range: 30.94 to 142.33 GPa
Fitted function: Gaussian

Results
x0 = 4.21258e+10 ± 2.80004e+07 Pa
y0 = 2.09363e-12 ± 8.36837e-13 Pa^-1
a = 3.20628e-10 ± 8.18998e-12 Pa^-1
b = 1.34623e+09 ± 3.99205e+07 Pa

Residual sum: 9.16123e-23

Correlation matrix
1.000
-0.000 1.000
-0.000 -0.072 1.000
0.000 -0.125 -0.562 1.000

Figure S3. Representative fitting process for obtaining height distribution and modulus distribution of the PFQNM maps of films. And the representative Gaussian fitting results of height and modulus distribution for a Mxene film.

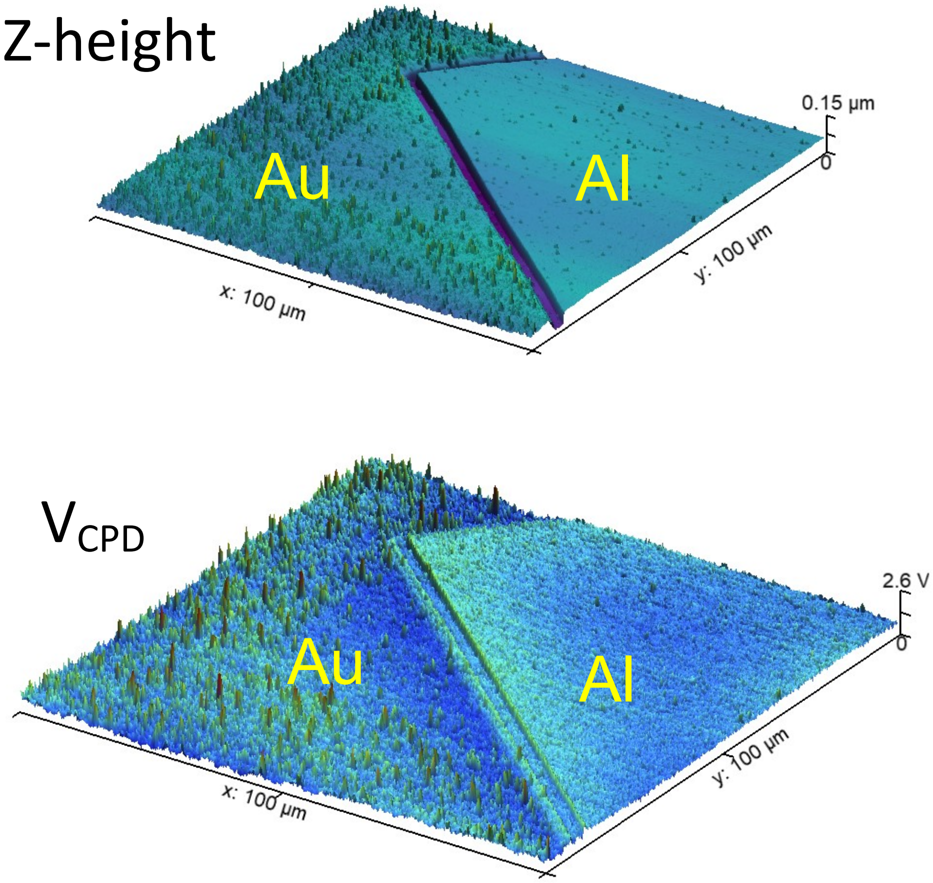

Figure S4. Representative KPFM images for the Au-Al reference sample for KPFM PtIr tip calibration.

The calibrated PtIr probe was then used to map the local variation of the absolute CPD over the Mxene and its composites. The absolute and reproducible Φ values were obtained as reported in Figure 3, and the numbers are listed in the Table below

Materials	Mxene	Mxene noPIL-30 wt.% HCNF	Mxene PIL-30 wt.% HCNF	Mxene PIL-40 wt.% HCNF
Φ (eV)	4.70±0.02	1.70±0.03	1.34±0.00	0.77±0.00

The nanocellulose specimens were prepared using the original suspensions at a concentration of 0.12 g·L^{-1}, without dilution. The mica surface was fully covered by HCNFs, and the PFQNM results are comparable to those obtained from the film measurements. The dense coverage of HCNF layers on the mica surface eliminated the uncertainty typically associated with measuring nanomechanical and adhesion forces on individual fibers.

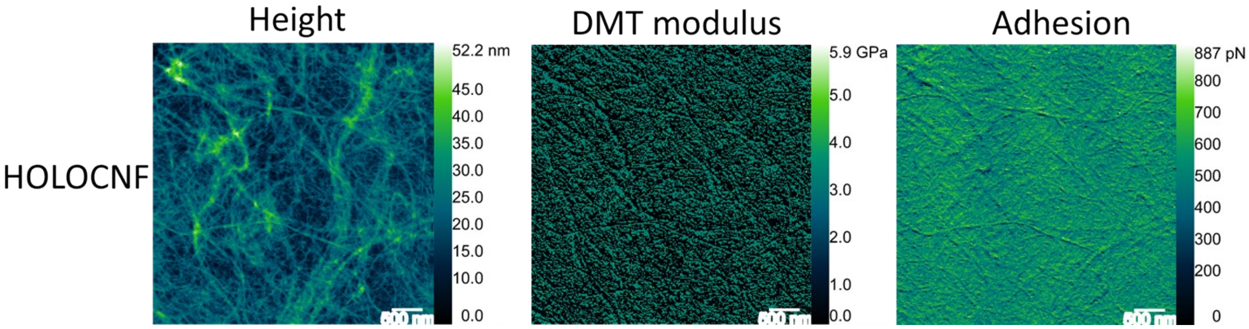

Figure S5. Representative PFQNM images of HCNF layers immobilized on a mica surface, along with statistical analyses of the DMT modulus, adhesion force, and height distribution.

Experimental

Detailed preparation method of films are provided as follows:

Determination of molecular weight of the synthesized PIL

Poly(1-cyanomethyl-3-vinylimidazolium bromide) (PIL, average M_w = 118 500) was synthesized according to a reported method. [1] Hydrophilic polyvinylidene fluoride membranes (PVDF membrane, pore size 0.22 µm) were purchased from Sigma-Aldrich. Molar masses (M_n and M_w) and dispersity ($Đ$) of PIL were characterized by size exclusion chromatography (SEC) at 25 °C in aqueous sodium acetate (0.1 M)/acetic acid (0.1 M) buffer (pH = 4.5) (DMF) containing MeOH (20 vol%) at a flow rate of 1 mL min^{-1} with a poly(2-vinylpyridine) calibration. SEC curves were recorded with an Agilent 1260 Infinity II equipped with two columns (PSS NOVEMA Max analytical Linear S and XL), a variable wavelength detector and refractive index detectors.

Preparation of water-stable $Ti_3C_2T_X$ ((MXene) colloidal dispersion

$Ti_3C_2T_X$ (MXene) was synthesized by etching Ti_3AlC_2 (MAX) powder with a mixture of LiF and HCl solution. 2.5 g of LiF was dissolved in 30 mL of 9 M HCl in water, followed by the slow addition of 1.5 g of Ti_3AlC_2 (400 mesh) powder. The mixture was stirred at 500 rpm for 24 h at 35 °C. Upon completion of the reaction, the mixture was diluted with 200 mL of deionized water and centrifuged at 12 000 rpm for 1 h. The upper supernatant was decanted to retain the sediment. This washing process was repeated 4 times to obtain a slurry sediment. The slurry sediment was then dispersed in deionized water by shaking using a Vortex-Genie 2 mixer for 20 min, followed

by centrifugation at 12 000 rpm for 1 h. The supernatant was discarded. This process was repeated 5 times to obtain successfully exfoliated $Ti_3C_2T_X$. The as-exfoliated $Ti_3C_2T_X$ in water was sonicated under a nitrogen-protective atmosphere for 10 min and then centrifuged at 3000 rpm for 20 min to remove the unexfoliated $Ti_3C_2T_X$ particles. The yield of the $Ti_3C_2T_X$ at 35 °C was 30.4 ± 4.0%.

Preparation of PIL-modified MXene flake colloidal dispersion

PIL-MXene were prepared as follows, 75 milligrams of 5 mg mL^{-1} MXene dispersion was added to 40 mL of a 3.5 mg mL^{-1} PIL solution dropwise, stirred for 5 min and then centrifuged twice at 12 000 rpm to get rid of the unbound polymers in the system.

Preparation of Mxene, PIL-Mxene and HCNF-MXene films

To prepare the $Ti_3C_2T_x$ (MXene) film, 10 mL of a 1 mg·mL^{-1} $Ti_3C_2T_x$ dispersion was vacuum-filtered onto a PVDF membrane to form the first layer. To prepare the PIL-MXene film, a second layer was formed by applying 1 mL of a 5 mg·mL^{-1} PIL-modified $Ti_3C_2T_x$ dispersion on top of the $Ti_3C_2T_x$ layer. To prepare the HCNF-MXene and HCNF-PIL-MXene films, suspensions containing 30 wt% and 40 wt% HCNFs, respectively, were subsequently applied onto the MXene dispersions.

The resulting films were then peeled off from the PVDF membrane for further characterisation. The actuation speed was tested using rectangular strips measuring 3 mm × 30 mm, cut from the prepared films, and evaluated at 20 °C in acetone vapor (saturated vapour pressure, 24.478 kPa). The strips were cut at approximately 45° relative to the anisotropic axis of the film, as indicated by the line pattern on the PVDF membrane. [2]

The X-ray diffraction XRD, X-ray photoelectron spectroscopy (XPS), Raman spectroscopy, Scanning electron microscopy (SEM) and Transmission electron microscopy (TEM) data have been provided below. [3,4] The successful etching and exfoliation were further confirmed by XRD patterns (Fig. S6 (a) below). The characteristic peaks corresponding to the (101), (104), and (105) planes of the MAX phase at 34.1°, 39.1°, and 41.8°, respectively, disappeared after the etching process. Correspondingly, oxygen (O), hydroxyl (OH) groups (from H_2O), and fluorine (F) (from LiF) became bound to the titanium (Ti) sites instead of the removed Al in Ti_3AlC_2 (MAX). This resulted in MXene nanosheets with a uniform distribution of titanium (Ti), carbon (C), oxygen (O), and fluorine (F) elements.

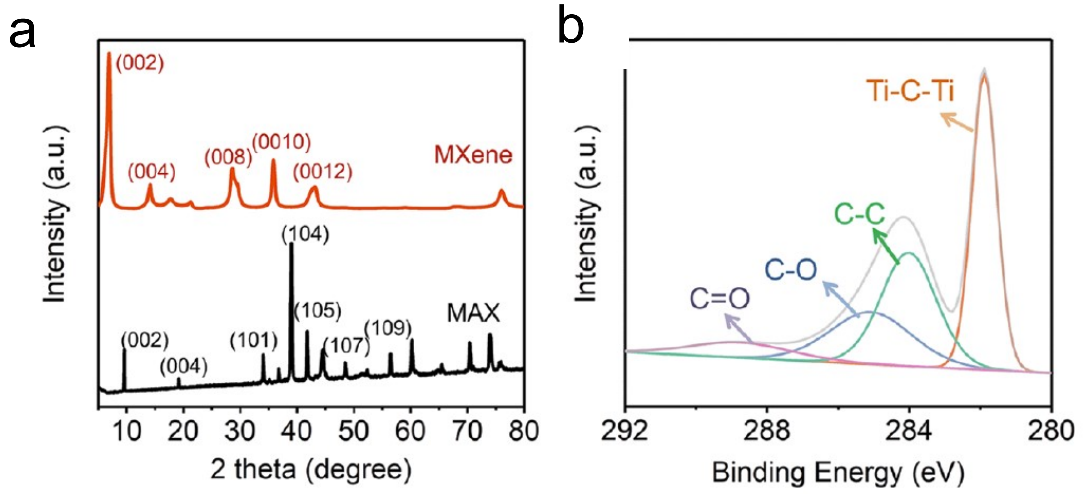

Figure S6. (a) XRD patterns of the Ti_3AlC_2 (MAX) and as-prepared MXene nanosheets. (b) Fitted XPS spectra of C 1s of MXene nanosheets. The XPS analysis suggests that the C $_{1s}$ spectra of MXene nanosheets could be fitted with four components corresponding to Ti–C–Ti (281.9 eV), C–C (284.1 eV), C–O (285.2 eV), and C=O (288.9 eV).

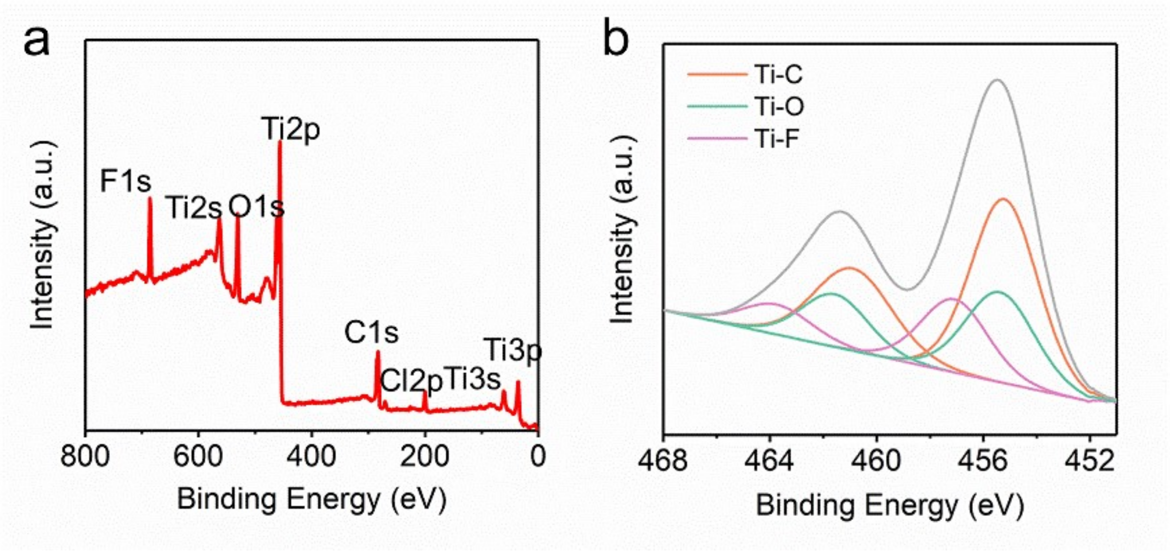

Figure S7. (a) XPS spectrum of MXene nanosheets. (b) Fitted XPS spectra of Ti 2p of MXene nanosheets corresponding to Ti-C (455.2 and 461.0 eV), Ti-O (455.5 and 461.7 eV), and Ti-F (457.2 and 463.8 eV). (data reused from ref., [4])

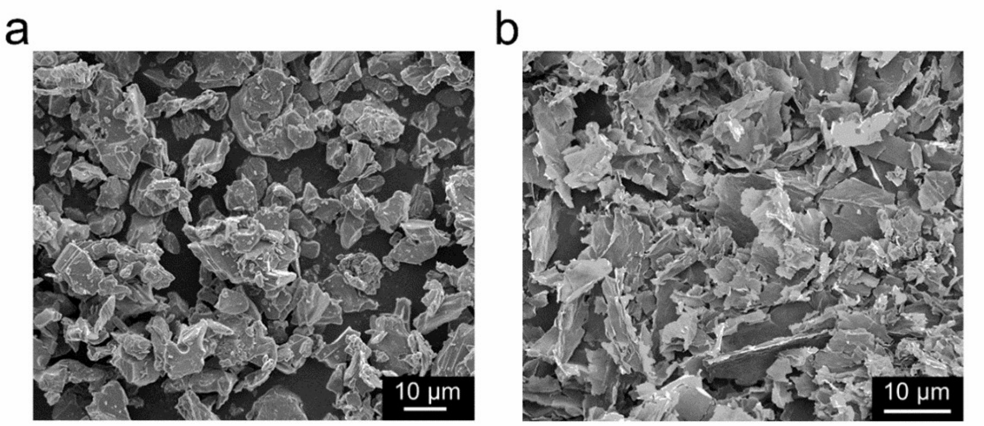

Figure S8. The SEM images of (a) Ti_3AlC_2 (MAX) powder and (b) MXene nanosheets. (data reused from ref., [4])

The uniform distribution of titanium (Ti), carbon (C), oxygen (O), and fluorine (F) elements was verified by energy-dispersive X-ray spectroscopy (EDS) elemental mapping results (see Fig. S9 below)

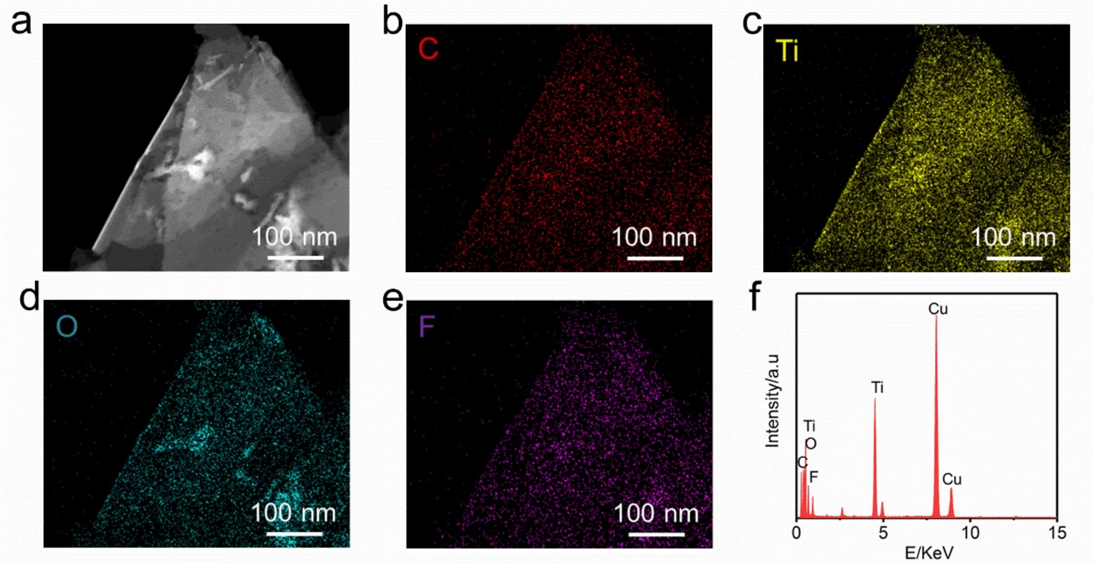

Figure S9. (a) TEM image of MXene nanosheets. EDS mapping images of MXene nanosheet showing (b) C, (c) Ti, (d) O, and (e) F elements. (f) The corresponding element pattern of MXene nanosheet. (data reused from ref., [4])

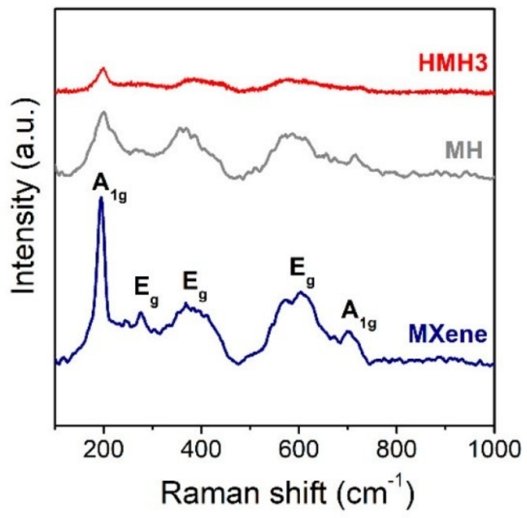

Figure S10. The Raman spectrum of pure MXene showed characteristic Raman modes centered at 195.2 and 702 cm^{-1}, which are ascribed to the A_{1g} symmetric out-of-plane vibrations of Ti and C atoms, respectively. Additionally, the Raman bands observed at 276.6, 367.9, and 603.6 cm^{-1} are attributed to the in-plane vibrations (E_g group) of Ti, C, and atoms in the surface functional groups. (data reused from ref., [5])

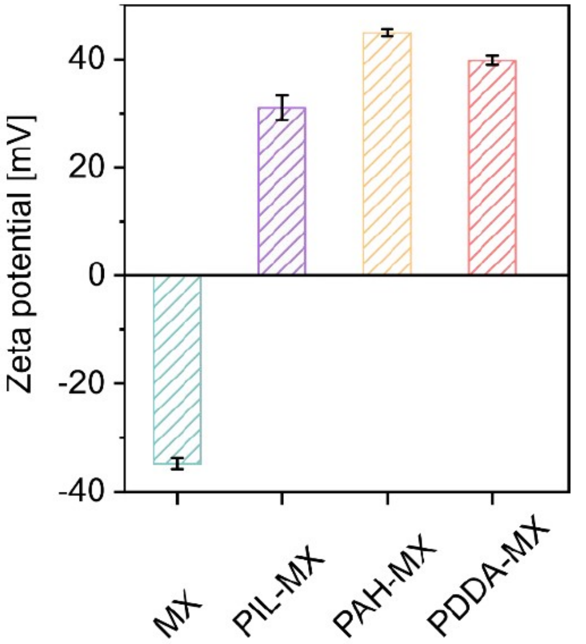

Figure S11. Zeta potentials of MXene (MX) and PIL-modified MXenes in water. (data reused from ref. [2])

References

1 M. Zhang, K. Dong, S. Saeedi Garakani, A. Khorsand Kheirabad, I. Manke, M. Wu, H. Wang, L. Qu and J. Yuan, *Adv. Sci.*, DOI:10.1002/advs.202105072.

2 A. Di, C. Wang, Y. Wang, H. He, W. Deng, P. Stiernet, L. Bergström, J. Yuan and M. Zhang, *Chem. Sci.*, 2025, **16**, 2191–2201.

3 X. Yang, M. S. Reid, P. Olsén and L. A. Berglund, *ACS Nano*, 2020, **14**, 724–735.

4 J. Chang, B. Pang, H. Zhang, K. Pang, M. Zhang and J. Yuan, *Adv. Fiber Mater.*, 2024, **6**, 252–263.

5 A. Sikdar, F. Héraly, H. Zhang, S. Hall, K. Pang, M. Zhang and J. Yuan, *ACS Nano*, 2024, **18**, 3707–3719.
